# Supplementary material for: Antibiotic Susceptibility Patterns and Virulence-Associated Factors of Vancomycin-Resistant Enterococcal Isolates from Tertiary Care Hospitals
Source: Antibiotics (Basel). 2023 May 29;12(6):981. doi: 10.3390/antibiotics12060981 (PMC10295198; doi:10.3390/antibiotics12060981)
Supplement: Supplementary file 1 [file antibiotics-12-00981-s001.zip › antibiotics-2379922-supplementary.pdf]

Table S1. Details of primers used to amplify the virulence genes (*gelE, sprE, esp, ace, asa, hyl, cylA, vanA, vanB*) from vancomycin resistant enterococcal isolates

Target Gene	Virulence factor	5′- 3′ Sequence	Product size (bp)	Reference
gelE	Gelatinase	AAGAAAAAGAAGTAGACCAAC<br>AAACGGCAAGACAAGTAAATA	400	Eaton and Gasson (2000)
sprE	Serine proetase	CTGAGGACAGAAGACAAGAAG<br>GGTTTTTCTCACCTGGATAG	432	Eaton and Gasson (2000)
esp	Enterococcal surface protein	TTGCTAATGCTAGTCCACGACC<br>GCGTCAACACTTGCATTGCCGAA	930	Shankar et al. (1999)
ace	Collagen adhesion	CAGGCCAACATCAAGCAACA<br>GCTTGCCTCGCCTTCTACAA	125	Haghi et al. (2019)
asaI	Aggregation substance	GCACGCTATTACGAACTATGA<br>TAAGAAAGAACATCACCACGA	375	Vankerckhoven et al (2004)
hyl	Hyaluronidase	ACAGAAGAGCTGCAGGAAATG<br>GACTGACGTCCAAGTTTCCAA	276	Haghi et al. (2019)
cylA	Cytolysin CylA	TGGATGATAGTGATAGGAAGT<br>TCTACAGTAAATCTTTCGTCA	517	Eaton and Gasson (2000)
vanA	Vancomycin A resistance	CATGAATAGAATAAAAGTTGCAATA<br>CCCCTTTAACGCTAATACGTCAA	1030	Ghalandarzadeh et al (2013)
vanB	Vancomycin B resistance	GTGACAAACCGGAGGCGAGGA<br>CCGCCATCCTCCTGCAAAAAA	433	Ghalandarzadeh et al (2013)

Table S2. Phenotypic and genotypic profile of virulence factors associated with vancomycin resistant *E. faecalis* isolates

	Phenotypic virulence factors						Genotypic virulence factors							Vancomycin resistance gene	
***E. faecalis* (no=37)**	**Gelatinase**	**Protease**	**Hemolysis**	**Biofilm assay OD_{570}**	**Biofilm index**	**Biofilm**	***gelE***	***sprE***	***esp***	***ace***	***asaI***	***hyl***	***cylA***	***vanA***	***vanB***
Efl1	P	P	P	0.171	3	S	P	P	P	P	P	N	P	P	N
Efl2	P	P	P	0.182	3	S	N	N	P	P	P	N	P	P	N
Efl3	P	P	P	0.167	3	S	P	P	P	P	P	N	P	P	N
Efl4	P	P	P	0.083	2	M	P	P	N	N	P	N	N	P	N
Efl5	P	P	P	0.158	3	S	P	P	P	P	P	N	N	P	N

Efl6	P	N	P	0.168	3	S	P	P	P	P	P	N	N	P	N
Efl7	P	P	P	0.094	2	M	P	P	P	P	P	N	N	P	N
Efl8	N	N	P	0.021	0	N	N	N	N	N	N	N	P	P	N
Efl9	P	P	P	0.148	3	S	P	P	N	N	N	N	N	P	N
Efl10	N	N	N	0.021	0	N	N	N	N	N	N	N	N	N	N
Efl11	N	N	N	0.028	0	N	N	N	N	N	N	N	N	P	N
Efl12	P	N	N	0.023	0	N	P	P	N	N	N	N	N	P	N
Efl13	P	N	N	0.041	1	W	P	N	N	N	N	N	N	P	N
Efl14	P	N	P	0.089	2	M	P	P	P	P	P	N	P	P	N
Efl15	P	P	P	0.051	1	W	N	N	P	P	P	N	N	N	N
Efl16	P	P	P	0.193	3	S	P	N	P	P	P	N	N	P	N
Efl17	P	P	P	0.172	3	S	P	P	P	P	P	N	N	P	N
Efl18	P	P	P	0.171	3	S	P	P	P	P	P	P	P	P	N
Efl19	P	P	P	0.082	2	M	P	P	P	P	P	N	P	P	N
Efl20	P	P	P	0.091	2	M	P	P	P	P	P	N	P	P	N
Efl21	P	P	P	0.198	3	S	P	P	P	P	P	N	P	P	N
Efl22	P	P	P	0.181	3	S	P	P	P	P	P	N	P	P	N
Efl23	N	N	P	0.117	2	M	N	N	N	N	P	N	P	P	N
Efl24	P	P	P	0.061	1	W	P	N	N	N	N	N	P	P	N
Efl25	P	N	P	0.184	3	S	P	P	P	P	P	N	P	N	N
Efl26	P	P	P	0.192	3	S	P	P	N	N	N	N	P	P	N
Efl27	P	P	P	0.185	3	S	P	N	P	P	P	N	P	P	N
Efl28	P	P	P	0.081	2	M	P	P	P	P	P	P	P	P	N
Efl29	P	P	P	0.189	3	S	P	P	P	P	P	N	P	P	N
Efl30	N	P	P	0.194	3	S	N	N	P	P	P	N	P	P	N
Efl31	N	P	P	0.013	0	N	N	N	P	N	N	N	P	P	N
Efl32	P	P	P	0.187	3	S	P	P	P	P	P	N	N	P	N
Efl33	P	P	P	0.156	3	S	P	P	P	P	P	N	P	P	N
Efl34	P	P	P	0.081	2	M	P	P	P	P	P	N	P	P	N
Efl35	P	P	P	0.094	2	M	N	N	N	N	N	N	P	N	N
Efl36	P	P	P	0.098	2	M	P	P	P	P	P	N	P	P	N
Efl37	P	P	P	0.176	3	S	P	P	P	P	P	N	N	P	N

P: Positive, N: Negative, S: Strong, M: Moderate, W: Weak

Biofilm index is projected based on **0**: $OD_T \leq OD_C$, **1**: $OD_C < OD_T \leq 2X\ OD_C$, **2**: $2X\ OD_C < OD_T \leq 4X\ OD_C$, **3**: $OD_T > 4X\ OD_C$ (where OD_T is the OD of test sample; OD_C is the OD of the negative control [45,46], $OD_C = 0.036$, $2X\ OD_C = 0.072$, $4X\ OD_C = 0.144$

Table S3. Phenotypic and genotypic profile of virulence factors associated with vancomycin resistant *E. faecium* isolates.

E.faecium (no=54)	Phenotypic virulence factors						Genotypic virulence factors							Vancomycin resistance gene	
	Gelatinase	Protease	Hemolysis	Biofilm assay OD_{570}	Biofilm	Biofilm	*gelE*	*sprE*	*esp*	*ace*	*asaI*	*hyl*	*cylA*	*vanA*	*vanB*
Efae1	N	N	P	0.012	0	N	N	N	N	N	N	N	P	P	N
Efae2	N	N	N	0.018	0	N	N	N	N	N	N	N	N	N	N
Efae3	P	P	P	0.197	3	S	P	P	P	P	P	P	P	P	N
Efae4	P	P	P	0.098	2	M	P	N	P	P	P	N	P	P	N
Efae5	P	P	P	0.089	2	M	P	P	P	P	P	N	P	P	N
Efae6	P	P	P	0.208	3	S	P	P	P	P	P	P	P	P	N
Efae7	N	N	N	0.081	2	M	N	N	P	P	P	N	N	N	N
Efae8	N	P	P	0.011	0	N	N	P	N	N	P	N	P	P	N
Efae9	P	N	P	0.080	2	M	P	N	P	P	P	N	P	P	N
Efae10	P	N	N	0.213	3	S	N	N	P	P	P	P	N	P	N
Efae11	P	N	P	0.197	3	S	P	N	P	P	P	N	P	P	N
Efae12	P	P	P	0.191	3	S	P	P	P	P	P	N	P	P	N
Efae13	P	N	N	0.196	3	S	P	N	P	P	P	N	N	P	N
Efae14	N	P	N	0.019	0	N	N	P	N	N	N	P	N	P	N
Efae15	P	P	P	0.081	2	M	N	P	P	P	P	P	P	P	N
Efae16	P	P	P	0.091	2	M	P	P	P	P	P	N	P	P	N
Efae17	N	P	P	0.016	0	N	N	P	N	N	N	N	P	P	N
Efae18	P	P	P	0.112	2	M	P	P	P	P	P	N	P	P	N
Efae19	N	N	N	0.021	0	N	N	N	N	N	N	N	N	N	N
Efae20	N	P	N	0.016	0	N	N	P	N	N	N	N	N	P	N
Efae21	P	P	P	0.0977	2	M	P	P	P	P	P	N	P	P	N
Efae22	P	P	P	0.891	2	M	P	P	P	P	P	N	P	P	N
Efae23	P	P	P	0.102	2	M	P	P	P	P	P	N	P	P	N
Efae24	P	N	N	0.023	0	N	P	N	N	N	N	N	N	P	N
Efae25	P	N	N	0.020	0	N	P	N	N	N	N	N	N	P	N
Efae26	N	N	N	0.015	0	N	N	N	N	N	N	N	N	P	N
Efae27	P	N	N	0.020	0	N	P	N	N	N	N	N	N	N	N
Efae28	P	N	P	0.010	0	N	P	N	N	N	N	P	P	P	N
Efae29	N	P	N	0.157	3	S	N	P	P	P	P	P	N	P	N
Efae30	P	N	N	0.013	0	N	P	N	N	N	N	N	N	P	N
Efae31	P	P	P	0.172	3	S	P	P	P	P	P	P	P	P	N

Efae32	P	P	P	0.198	3	S	P	P	P	P	P	P	P	P	N
Efae33	P	N	N	0.012	0	N	P	N	N	N	N	N	N	P	N
Efae34	N	P	N	0.010	0	N	N	P	N	N	N	P	N	P	N
Efae35	P	P	P	0.051	1	W	P	N	N	N	P	P	P	P	N
Efae36	P	P	P	0.013	0	N	P	P	N	N	N	P	P	P	N
Efae37	P	P	P	0.020	0	N	P	N	N	N	N	P	P	P	N
Efae38	P	N	N	0.047	1	W	P	N	P	P	P	N	N	P	N
Efae39	P	N	P	0.109	2	M	P	N	P	N	N	P	N	P	N
Efae40	P	N	N	0.098	2	M	N	N	P	P	P	N	N	P	N
Efae41	P	P	P	0.178	3	S	P	P	P	P	P	P	P	P	N
Efae42	P	N	N	0.192	3	S	P	N	P	P	P	P	P	P	N
Efae43	P	N	N	0.969	2	M	P	N	P	P	P	P	N	P	N
Efae44	P	N	N	0.168	3	S	P	N	P	N	N	N	P	P	N
Efae45	P	P	P	0.859	2	M	P	P	P	P	P	N	N	P	N
Efae46	P	P	N	0.043	1	W	P	P	P	P	P	N	N	P	N
Efae47	P	N	N	0.052	1	W	P	N	P	N	N	N	N	P	N
Efae48	P	P	P	0.049	1	W	P	P	P	N	N	N	P	P	N
Efae49	N	P	N	0.013	0	N	N	N	N	N	N	N	N	P	N
Efae50	P	P	P	0.191	3	S	P	N	P	P	P	N	P	P	N
Efae51	P	P	P	0.051	1	W	P	P	N	N	N	N	P	P	N
Efae52	P	N	N	0.043	1	W	P	N	P	P	P	N	N	P	N
Efae53	P	P	P	0.180	3	S	N	P	P	P	P	N	P	P	N
Efae54	P	P	P	0.171	3	S	N	P	P	P	P	N	P	P	N

P: Positive, N: Negative, S: Strong, M: Moderate, W: Weak

Biofilm index is projected based on **0**: $OD_T \leq OD_C$, **1**: $OD_C < OD_T \leq 2X\ OD_C$, **2**: $2X\ OD_C < OD_T \leq 4X\ OD_C$, **3**: $OD_T > 4X\ OD_C$ (where OD_T is the OD of test sample; OD_C is the OD of the negative control [45,46], OD_C= 0.036, 2X OD_C= 0.072, 4X OD_C=0.144

References

45. Stepanović, S.; Vuković, D.; Dakić, I.; Savić, B.; Švabić-Vlahović, M. A modified microtiter-plate test for quantification of staphylococcal biofilm formation. J. Microbiol. Methods. 2000, 40, 175–179. https://doi.org/10.1016/s0167-7012(00)00122-6.
46. Cui, P.; Feng, L.; Zhang, L.; He, J.; An, T.; Fu, X.; Li, C.; Zhao, X.; Zhai, Y.; Li, H.; et al. Antimicrobial resistance, virulence genes, and biofilm formation capacity among enterococcus species from yaks in Aba Tibetan Autonomous Prefecture, China. Front. Microbiol. 2020, 11, 1250. https://doi.org/10.3389/fmicb.2020.01250
